# The “Picasso faces” of diabetic kidney disease – how the art of phenotyping and molecular biomarkers is transforming clinical nephrology: an observational study in patients with type 2 diabetes

**DOI:** 10.1186/s12882-025-04592-4

**Published:** 2025-11-19

**Authors:** Małgorzata Rodzoń-Norwicz, Natalia Potocka, Marzena Skrzypa, Izabela Zawlik, Katarzyna Milian–Ciesielska, Patryk Kogut, Krzysztof Gargasz, Michael Maes, Magdalena Sowa-Kućma, Agnieszka Gala-Błądzińska

**Affiliations:** 1 Faculty of Medicine, Department of Human Physiology, University of Rzeszów, al. Tadeusza Rejtana 16C, Rzeszów, 35-959 Poland; 2 Clinic of Internal Medicine, Nephrology and Endocrinology with Nuclear Medicine Laboratory and Dialysis Center, State Hospital 2 in Rzeszów, Lwowska Street 60, Rzeszów, 35-301, Poland; 3 Faculty of Medicine, Laboratory of Molecular Biology, Centre for Innovative Research in Medical and Natural Sciences, University of Rzeszów, al. Tadeusza Rejtana 16C, Rzeszów, 35-959 Poland; 4 Faculty of Medicine, Department of General Genetics, University of Rzeszów, al. Tadeusza Rejtana 16C, Rzeszów, 35-959 Poland; 5 Faculty of Medicine, Department of Pathomorphology, Jagiellonian University Medical College, Grzegorzecka 16, Krakow, 31-531 Poland; 6 Faculty of Medicine, Data Processing Laboratory, Natural and Medical Center for Innovative Research, University of Rzeszów, al. Tadeusza Rejtana 16C, Rzeszów, 35-959 Poland; 7 Faculty of Medicine, Department of Nephrology and Endocrinology, University of Rzeszów, al. Tadeusza Rejtana 16C, Rzeszów, 35-959 Poland; 8 Sichuan Provincial People’s Hospital, School of Medicine, Sichuan Provincial Center for Mental Health, University of Electronic Science and Technology of China, Chengdu, 610072 China; 9 Key Laboratory of Psychosomatic Medicine, Chinese Academy of Medical Sciences, Chengdu, 610072 China; 10 Department of Psychiatry, Faculty of Medicine, Chulalongkorn University and King Chulalongkorn Memorial Hospital, The Thai Red Cross Society, Bangkok, Thailand; 11 Department of Psychiatry, Medical University of Plovdiv, Plovdiv, Bulgaria; 12 Research Center, Medical University of Plovdiv, Plovdiv, Bulgaria; 13 Research and Innovation Program for the Development of MU - PLOVDIV – (SRIPD-MUP), Creation of a network of research higher schools, National Plan for Recovery and Sustainability, European Union – NextGenerationEU, Plovdiv, Bulgaria; 14 Kyung Hee University, 26 Kyungheedae-ro, Dongdaemun-gu, Seoul, 02447 Korea

**Keywords:** Diabetic kidney disease, Biomarkers, Phenotypes, Epigenetics

## Abstract

**Background and hypothesis:**

Diabetic kidney disease (DKD) in patients with type 2 diabetes mellitus (T2DM) presents heterogeneously, complicating risk assessment. This study evaluated microRNAs and other biomarkers for DKD phenotyping and predicting kidney function in clinical practice.

**Methods:**

Data from 79 patients with T2DM were analyzed. DKD phenotypes were defined based on eGFR and UACR:

• F1 (albuminuric): eGFR < 60 mL/min/1.73 m², UACR ≥ 300 mg/g.

• F2 (non-albuminuric, preserved filtration): eGFR ≥ 60 mL/min/1.73 m², UACR ≤ 30 mg/g.

• F3 (non-albuminuric, reduced filtration): eGFR < 60 mL/min/1.73 m², UACR ≤ 30 mg/g.

• F4 (moderately increased albuminuria): UACR > 30 and < 300 mg/g was shown for completeness but excluded from primary analyses.

Multiple regression and partial least squares structural equation modeling (PLS-SEM) were applied to identify predictors of eGFR. Discriminant analysis (including age, ACE, uric acid, and AIP) was used for phenotype classification. Serum levels of hsa-miR-126-3p and hsa-miR-423-5p were compared between phenotypes.

**Results:**

Independent predictors of eGFR included ACE (β = − 0.478; *p* < 0.001), age (β = − 0.336), AIP (β = − 0.245), and hsa-miR-423-5p (β = 0.138; *p* = 0.033) (R² = 0.619). In the PLS-SEM model, ACE, AIP, and hsa-miR-423-5p had significant direct effects on eGFR, while ACE was modulated by hsa-miR-126-3p, age, and BMI. Discriminant analysis correctly classified 87.5% of patients (Wilks’ Lambda, *p* < 0.05). F1 exhibited the highest ACE and hsa-miR-126-3p levels, lowest HDL-C, and most microvascular complications. F2 had the best renal function, lowest ACE and miR-126-3p expression, and the highest proportion of women. F3 patients were the oldest, with elevated uric acid and hsa-miR-423-5p levels. Coronary heart disease was most common in F1 and F3, while stroke occurred only in F1 and F2.

**Conclusions:**

ACE, AIP, and the miRNAs hsa-miR-126-3p and hsa-miR-423-5p may support DKD phenotyping and kidney function prediction. Incorporating these markers into clinical models could enable the implementation of individualized nephroprotective strategies in patients with T2DM.

## Introduction

Diabetes mellitus (DM) remains one of the major global health challenges. According to the International Diabetes Federation (IDF), the prevalence of type 2 diabetes mellitus (T2DM) in the adult population continues to rise - from the current 10.5% to a projected 12% by 2045 [1]. Diabetic kidney disease (DKD), which affects approximately 30–40% of individuals with DM [2, 10], is a leading cause of chronic kidney disease (CKD) and significantly increases the risk of cardiovascular complications and mortality.

**Fig. 1:**
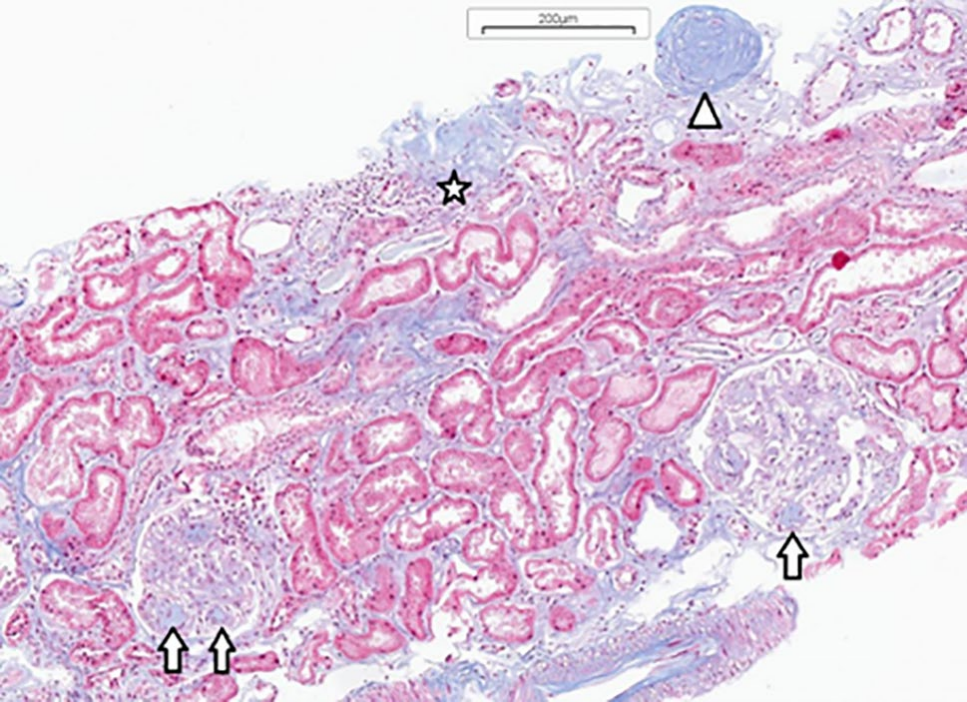
Diabetic nephropathy. Globally sclerosed glomerulus (triangle), glomeruli with Kimmelstiel-Wilson nodules (arrows) and area of tubular atrophy, mild chronic interstitial inflammation and fibrosis (star) (trichrome stain, x100). Kidney biopsy specimen obtained from one of the study participants – authors’ own material

According to the Kidney Disease: Improving Global Outcomes (KDIGO) guidelines, DKD is a clinical diagnosis made in a patient with DM and CKD, based on an estimated glomerular filtration rate (eGFR) and the urinary albumin-to-creatinine ratio (UACR) measured in a spot urine sample. In contrast, the term diabetic nephropathy (DN) refers specifically to histopathological lesions characteristic of DM, confirmed by kidney biopsy [3] (Fig. 1).

Despite advances in treatment, the prevalence of DKD has not declined over the past three decades. Therefore, early identification of patients at high risk of progression remains crucial, especially given the growing recognition of the clinical heterogeneity of DKD. The classical DKD phenotype, described by Mogensen as phenotype 1 (F1), is characterized by increasing albuminuria and a progressive decline in eGFR, and is most commonly observed in patients with type 1 diabetes mellitus (T1DM) and poor glycaemic control [4]. However, studies by Królewski et al. have shown that albuminuria is not always a predictor of kidney function decline [5].

It is now recognized that DKD may present with distinct clinical phenotypes [6]. In addition to the classical F1 phenotype, other subtypes include phenotype 2 (F2), in which albuminuria regresses or is absent despite kidney injury; phenotype 3 (F3), characterized by a rapid decline in eGFR without preceding albuminuria; and phenotype 4 (F4), marked by slow DKD progression without albuminuria [6]. Non-albuminuric phenotypes, increasingly observed in clinical practice, may involve a different pathogenesis, with predominant tubulointerstitial injury and fibrosis [7, 8].

Importantly, classical risk factors for progression - such as hypertension, hyperglycaemia and dyslipidaemia - do not fully account for the heterogeneous course of non-albuminuric forms of DKD [9–16]. At the same time, an increasing number of studies point to the role of epigenetic dysregulation and microRNAs (miRNAs) in the pathogenesis of DKD. Zapała et al. identified several miRNAs present in urinary extracellular vesicles (uEVs), including hsa-miR-375, hsa-miR-503 and hsa-miR-451a, which correlate with inflammation, apoptosis and tissue remodelling in DKD [17, 18].

Given the growing recognition of DKD heterogeneity, early and accurate phenotypic assessment may serve as an important prognostic indicator and enable personalized nephroprotective therapy already at the time of T2DM diagnosis. The aim of this study was to identify novel clinical and molecular biomarkers useful in differentiating DKD phenotypes.

## Materials and methods

### Study population

**Fig. 2:**
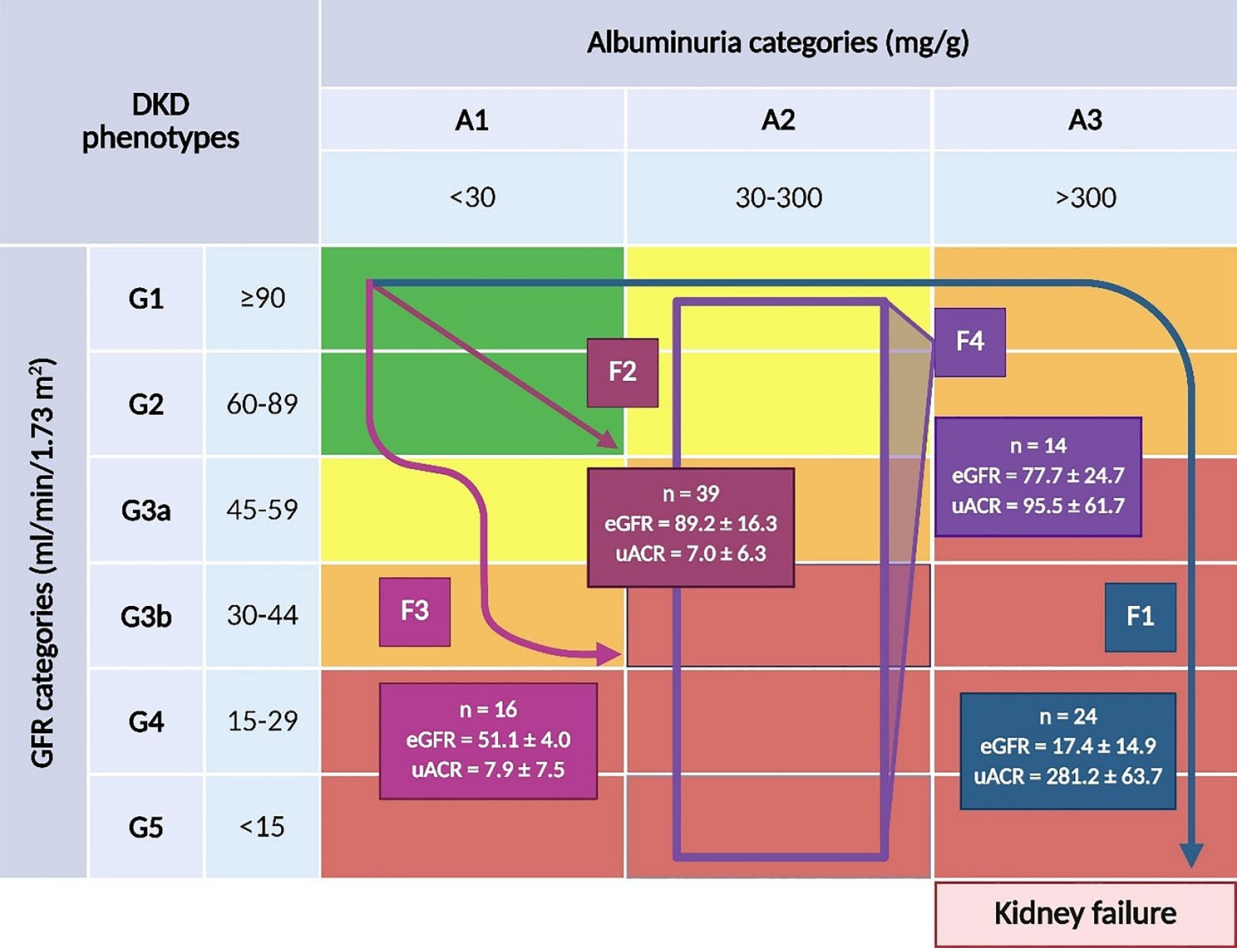
Classification of the study population into DKD phenotypes (F1–F4) based on eGFR and UACR according to KDIGO staging (G1–G5; A1–A3). Boxes report sample size (n) and mean ± SD for eGFR (mL/min/1.73 m²) and UACR (mg/g). Background colors indicate CKD risk categories; arrows depict observed trajectories. F4 (KDIGO A2, 30–300 mg/g) is shown descriptively and was excluded from primary analyses and from the discriminant model. Abbreviations: CKD – chronic kidney disease; DKD – diabetic kidney disease; eGFR – estimated glomerular filtration rate; KDIGO – Kidney Disease: Improving Global Outcomes; UACR – urinary albumin-to-creatinine ratio (created with BioRender.com)

A total of 97 patients with T2DM were prospectively recruited between December 1, 2018 and November 30, 2019 from the Nephrology Outpatient Clinic and Dialysis Unit of the Clinical Hospital. Clinical follow-up continued until June 30, 2023 (median 35 months). Seventy-nine patients (31 men, 48 women) were assigned by a nephrologist to one of three DKD phenotypes based on clinical assessment and eGFR/UACR values, using KDIGO eGFR (G1–G5) and albuminuria (A1–A3) categories [3] (Fig. 2):

- F1 (albuminuric): eGFR < 60 mL/min/1.73 m² and UACR ≥ 300 mg/g.
- F2 (non-albuminuric, preserved eGFR): eGFR ≥ 60 mL/min/1.73 m² and UACR ≤ 30 mg/g.
- F3 (non-albuminuric, reduced eGFR): eGFR < 60 mL/min/1.73 m² and UACR ≤ 30 mg/g.

Additionally, 14 patients had UACR 30–300 mg/g (KDIGO A2, “moderately increased” albuminuria [3]). For completeness, this subgroup is depicted as F4 in Fig. 2. It was treated as exploratory and was not included in the primary F1–F3 analyses or in the final discriminant model due to poor class separability and reduced model performance, as detailed in Results—Discriminant analysis. The remaining four patients did not meet the prespecified criteria and were excluded from primary phenotypic analyses. The diagnosis of DKD followed KDIGO 2022 guidelines. Because protocol renal biopsies were not performed in this outpatient cohort, disease severity was classified clinically, using eGFR and UACR, rather than by histology. Written informed consent was obtained from all participants. The study was conducted in accordance with the Declaration of Helsinki and was approved by the Bioethics Committee of the University of Rzeszów (approval no. 2018/06/10).

### Sample collection and laboratory procedures

#### Blood and urine collection

Venous blood was collected between 7:00 and 9:00 a.m. by trained nursing staff using standard procedures. Serum was obtained using S-Monovette^®^ clotting tubes (Sarstedt). After 30 min of incubation at room temperature, samples were centrifuged (10 min, 2500×g) and analyzed at the hospital’s certified laboratory. First-morning midstream urine was also used for analysis. Remaining biological material was biobanked at − 80 °C for later use in experimental assays.

#### miRNA expression analysis

miRNA was isolated from 300 µl of serum using the BioVendor miRNA Isolation Kit. Expression of hsa-miR-126-3p and hsa-miR-423-5p was assessed using miREIA immunoassays (BioVendor). The assay followed a standard protocol: hybridization, washing, HRP-conjugation, colorimetric development, and absorbance reading at 450 nm with reference at 630 nm using a Tecan reader. Absorbance was proportional to miRNA concentration.

#### Angiotensin-converting enzyme (ACE) and vascular endothelial growth factor A (VEGF-A) protein concentration

Serum ACE and VEGF-A levels were measured using enzyme-linked immunosorbent assay (ELISA) kits (Cloud-Clone Corp., USA). Absorbance was measured at 450 nm (Tecan Infinite 200 Pro), and concentrations were calculated from standard curves.

### Statistical analysis

Normality was assessed using the Shapiro-Wilk test. Due to non-normal distributions, the Kruskal-Wallis test with post hoc comparisons was used for continuous variables. Categorical variables were reported as absolute numbers and percentages and analyzed with the χ² test. Significance was set at *p* < 0.05. Analyses were performed in Statistica 13.3PL (StatSoft, Poland).

Spearman correlation coefficients were calculated to assess associations between renal indices (eGFR, UACR) and circulating miRNAs (hsa-miR-126-3p and hsa-miR-423-5p) in the overall cohort; phenotype-stratified correlations were considered exploratory. Statistical significance was assessed using two-sided tests with *p* < 0.05.

### Advanced modeling

PLS-SEM was applied to explore complex relations among independent (age, sex, miR-126-3p, miR-423-5p), mediating (ACE, atherogenic index of plasma [AIP]), and dependent (eGFR) variables. The model utilized a Partial Least Squares estimator and 5000-bootstrap resampling to test significance. Both direct and indirect effects on eGFR were evaluated.

To complement the structural model, a partial regression analysis was performed to further assess the relationship between ACE concentration and eGFR.

In addition, discriminant analysis was employed to classify patients into DKD phenotypes based on four predictors: AIP, age, ACE concentration, and uric acid level. Model significance and classification accuracy were assessed using standard statistical methods, including Wilks’ Lambda (*p* < 0.05).

An exploratory four-group model including the UACR 30–300 mg/g subgroup (F4) was evaluated; due to poor class separability and reduced classification accuracy, the final discriminant analysis is presented for F1-F3.

## Results

### Characteristics of the study population

A total of 97 patients were recruited, of whom 79 individuals with type 2 diabetes mellitus (T2DM) (mean age 67 ± 11.5 years) were included in the final analysis. Women constituted 60.8% of the study population. Obesity was prevalent, with a mean body mass index (BMI) of 31.4 ± 5.9 kg/m². The mean eGFR (CKD-EPI) was 59.7 ± 34.6 ml/min/1.73 m² (range 4–122), and the mean UACR was elevated (100.6 ± 158.9 mg/g). Full demographic, clinical, and laboratory characteristics are presented in Table 1.

**Table 1:** Selected demographic, clinical data and laboratory results for the research group

Variable	DKD (*n* = 79) Mean ± SD median (min-max)
Age [year]	66.73 ± 11.53; 67 (31–91)
Gender, female [n (%)]	48 (60.8%)
BMI [kg/m2]	31.4 ± 5.9; 31.6 (19.69–46.2)
ACE protein concentration [ng/ml]	581.63 ± 391.95; 564.31 (29.03–1406)
VEGF-A protein concentration [pg/ml]	459 ± 334.12; 402.17 (19.56-1820.3)
hsa-mir-126-3p [amol/µl]	0.52 ± 0.1; 0.58 (0.39–0.94)
hsa-mir-423-5p [amol/µl]	0.31 ± 0.14; 0.33 (0.06–0.48)
Creatinine [mg/dl]	2.03 ± 2.29; 0.95 (0.46–11.66)
eGFR _CKD-EPI_ [ml/min/1.73m^2^]	59.68 ± 34.63; 59 (4-122)
UACR mg/g	100.56 ± 158.87; 9.31 (0-882.34)
HbA1c	6.97 ± 1.63; 6.51 (5.2–15.9)
UNCR [ug/g]	23.3 ± 22.13; 15.96 (1.65-87)
Uric acid [mg/dl]	5.85 ± 1.31; 5.7 (3.3–10.1)
Total cholesterol [mg/dl]	174.3 ± 49.32; 167 (100–344)
LDL cholesterol [mg/dl]	96.88 ± 39.86; 89 (32–236)
HDL cholesterol [mg/dl]	47.25 ± 12.25; 47 (25–90)
TG [mg/dl]	150.12 ± 78.54; 123 (45–441)
AIP	0.47 ± 0.24; 0.45 (-0.03-0.95)

Abbreviations: ACE – angiotensin-converting enzyme; AIP – atherogenic index of plasma; BMI – body mass index; eGFR – estimated glomerular filtration rate; HbA1c – glycated hemoglobin; HDL-C – high-density lipoprotein cholesterol; hsa-miR – human microRNA; LDL-C – low-density lipoprotein cholesterol; TG – triglycerides; UACR – urinary albumin-to-creatinine ratio; UNCR – urinary nephrin-to-creatinine ratio; VEGF-A – vascular endothelial growth factor A

#### Comorbidities

Comorbid conditions included hypertension (88.8%), ischemic heart disease (29.6%), and heart failure (28.4%). Macroangiopathic complications were frequent: coronary artery disease (29.6%), ischemic stroke (11.1%), generalized atherosclerosis (49.2%), and amputations (7.4%). During the 35-month follow-up, seven patients (8.6%) died.

#### Pharmacotherapy and renal replacement therapy

Metformin was used in 59% of patients and insulin in 32%. Glycemic control was generally adequate (mean glycated hemoglobin [HbA1c] = 6.4 ± 0.7%). Statins were used by > 60% and ASA by 38%. Overall, 59.5% of patients (47/79) received RAAS blockade with ACE inhibitors or angiotensin receptor blockers (ARBs), with no significant differences between phenotypes (50.0% in F1, 60.5% in F2, and 70.6% in F3; *p* = 0.4). Chronic hemodialysis was required in 9.9%.

### Correlations between Circulating miRNAs and renal indices

In the overall cohort (*n* = 79), hsa-miR-423-5p correlated positively with eGFR (Spearman correlation coefficient = 0.23) and inversely with UACR (− 0.29). hsa-miR-126-3p correlated positively with UACR (0.24) and showed no statistically significant association with eGFR (− 0.10). Correlation magnitudes were small (from − 0.29 to 0.23), but the directions were biologically consistent. Detailed coefficients are reported in Table 2.

**Table 2:** Spearman correlation coefficients between eGFR, UACR and Circulating miRNAs (hsa-miR-126-3p and hsa-miR-423-5p) in the overall cohort. Bold values indicate statistical significance (two-sided *p* < 0.05)

	hsa-miR-126-3p (amoL/µl)	hsa-miR-423-5p (amoL/µl)
eGFR CKD-EPI (mL/min/1.73 m²)	-0.10	**0.23**
UACR (mg/g)	**0.24**	**-0.29**

Abbreviations: eGFR — estimated glomerular filtration rate; UACR — urine albumin-to-creatinine ratio; CKD-EPI — Chronic Kidney Disease Epidemiology Collaboration equation; hsa-miR — human microRNA

### Regression and structural modeling analysis

#### Multiple regression analysis

Model 1 (dependent variable: eGFR) explained 61.9% of variance (R² = 0.619; F(4,97) = 39.46; *p* < 0.001). ACE (β = −0.478; *p* < 0.001), age (β = −0.336; *p* < 0.001), and AIP (β = −0.245; *p* < 0.001) were inversely associated with eGFR, while hsa-miR-423-5p showed a positive effect (β = 0.138; *p* = 0.033).

Model 2 (dependent variable: ACE) explained 37.6% of the variance (R² = 0.376; F(3,99) = 20.06; *p* < 0.001). ACE was positively associated with age (β = 0.478; *p* < 0.001) and hsa-miR-126-3p (β = 0.165; *p* = 0.041), and negatively with BMI (β = −0.252; *p* = 0.002).

Model 3 (dependent variable: AIP) explained 15.6% of the variance (R² = 0.156; F(2,100) = 9.23; *p* < 0.001). Significant predictors included male sex (β = 0.311; *p* = 0.001) and age (β = 0.287; *p* = 0.002). Full results are shown in Table 3.

**Table 3:** Results of multiple regression analysis with eGFR, ACE and AIP levels as dependent variables

Dependent Variable	Explanatory Variable	β	T	*p* (predictor)	*R*²	F	Df	*p* (model)
eGFR (Model 1)					0.619	39.46	4/97	< 0.001
	ACE	–0.478	–6.42	< 0.001				
	Age	–0.336	–4.56	< 0.001				
	AIP	–0.245	–3.91	< 0.001				
	hsa-miR-423-5p	0.138	2.16	0.033				
ACE (Model 2)					0.376	20.06	3/99	< 0.001
	Age	0.478	5.97	< 0.001				
	BMI	–0.252	–3.16	0.002				
	hsa-miR-126-3p	0.165	2.07	0.041				
AIP (Model 3)					0.156	9.23	2/100	< 0.001
	Sex (male)	0.311	3.36	0.001				
	Age	0.287	3.10	0.002				

Multiple linear regression analyses showing predictors of eGFR, ACE protein levels, and Atherogenic Plasma Index (AIP). Model 1 (eGFR as the dependent variable) explains 61.9% of its variance, with significant negative associations for ACE, age, and AIP, and a positive association with hsa-miR-423-5p. Model 2 (ACE as the dependent variable) accounts for 37.6% of variance and identifies age and hsa-miR-126-3p as positive predictors, while BMI is negatively associated. Model 3 (AIP as the dependent variable) explains 15.6% of the variance and includes age and male sex as significant positive predictors. Variables without significant contribution (e.g., VEGF-A, BMI in model 1, and hsa-miR-126-3p in model 1) are not shown in the table. Abbreviations: ACE – angiotensin-converting enzyme; AIP – atherogenic index of plasma; BMI – body mass index; eGFR – estimated glomerular filtration rate; hsa-miR – human microRNA; β – standardized regression coefficient; T – t-statistic; p – significance level (p-value); R² – coefficient of determination; F – F-statistic; df – degrees of freedom

#### PLS-SEM model

**Fig. 3:**
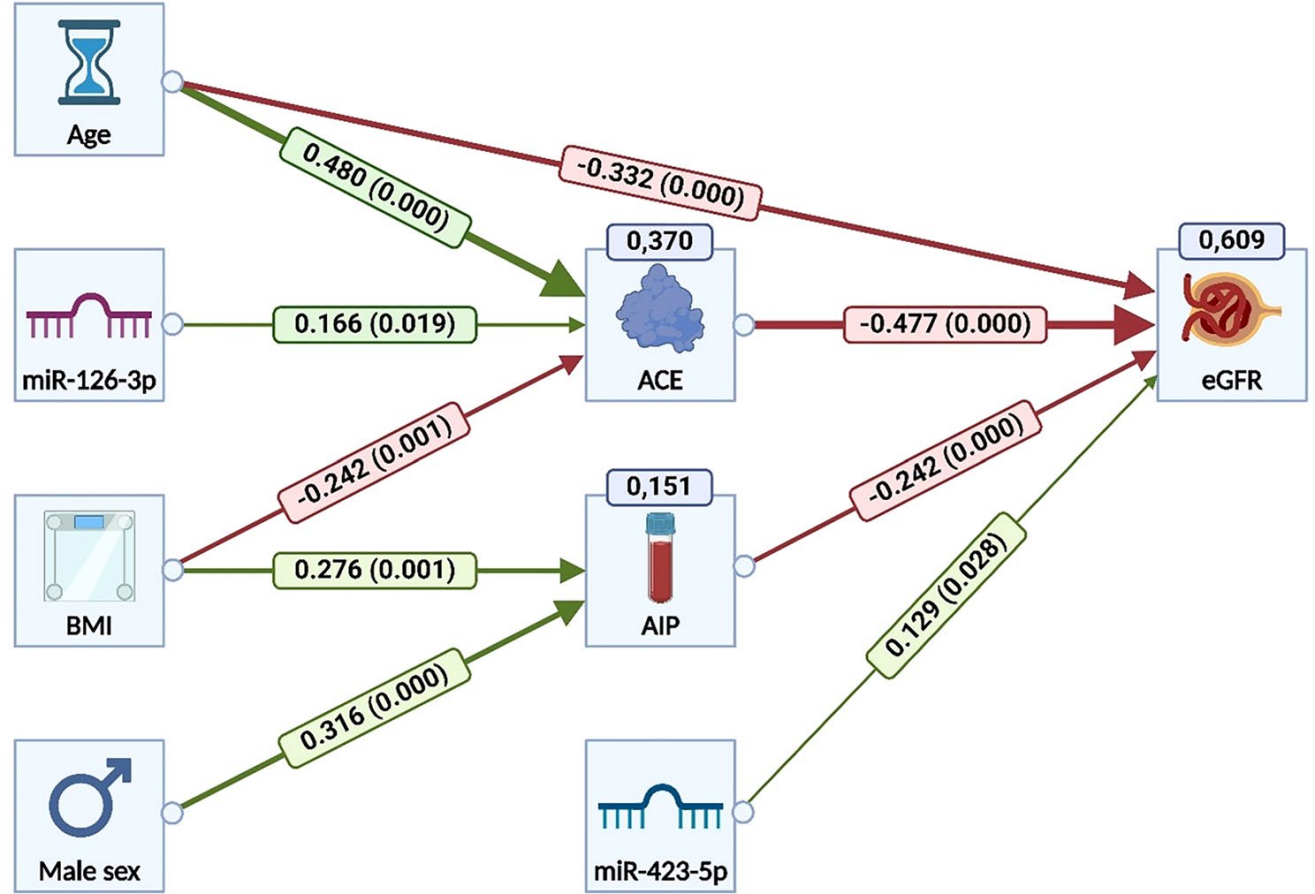
PLS path model showing all significant predictors of eGFR. The model demonstrates a strong overall fit (SRMR = 0.024). A total of 60.9% of the variance in eGFR was explained by age, ACE protein concentration, AIP, and hsa-miR-423-5p. Up to 37.0% of the variance in ACE was explained by age, hsa-miR-126-3p, and BMI, while 15.1% of the variance in AIP was explained by BMI and male sex. All specific indirect effects on eGFR via ACE or AIP were statistically significant for age (t = − 4.87, *p* < 0.001), BMI via AIP (t = − 2.17, *p* = 0.030) and via ACE (t = 3.04, *p* = 0.002), male sex (t = 2.57, *p* = 0.010), and hsa-miR-126-3p (t = 2.27, *p* = 0.023). However, the total indirect effect of BMI on eGFR was not statistically significant (t = 0.99, *p* = 0.323), due to opposing directional effects. In descending order of effect size, eGFR was most strongly predicted by age, ACE, AIP, male sex, hsa-miR-126-3p, and hsa-miR-423-5p. Abbreviations: ACE – angiotensin-converting enzyme; AIP – Atherogenic Index of Plasma; BMI – body mass index; eGFR – estimated glomerular filtration rate; SRMR – standardized root mean square residual; PLS – partial least squares; hsa-miR – human microRNA

The PLS-SEM model assessed direct and indirect effects on eGFR. It explained 60.9% of eGFR variance (standardized root mean square residual [SRMR] = 0.024), showing significant paths from ACE (t = 7.11; *p* < 0.001), age (t = 6.92; *p* < 0.001), AIP (t = 3.82; *p* < 0.001), male sex (t = − 2.57; *p* = 0.010), hsa-miR-126-3p (t = − 2.27; *p* = 0.023), and hsa-miR-423-5p (t = 2.19; *p* = 0.028). Indirect effects included: age (t = − 4.87; *p* < 0.001), BMI via AIP (t = − 2.17; *p* = 0.030), BMI via ACE (t = 3.04; *p* = 0.002), male sex (t = 2.57; *p* = 0.010), and hsa-miR-126-3p (t = 2.27; *p* = 0.023). The final PLS model is presented in Fig. 3.

#### Correlation between ACE and eGFR

**Fig. 4:**
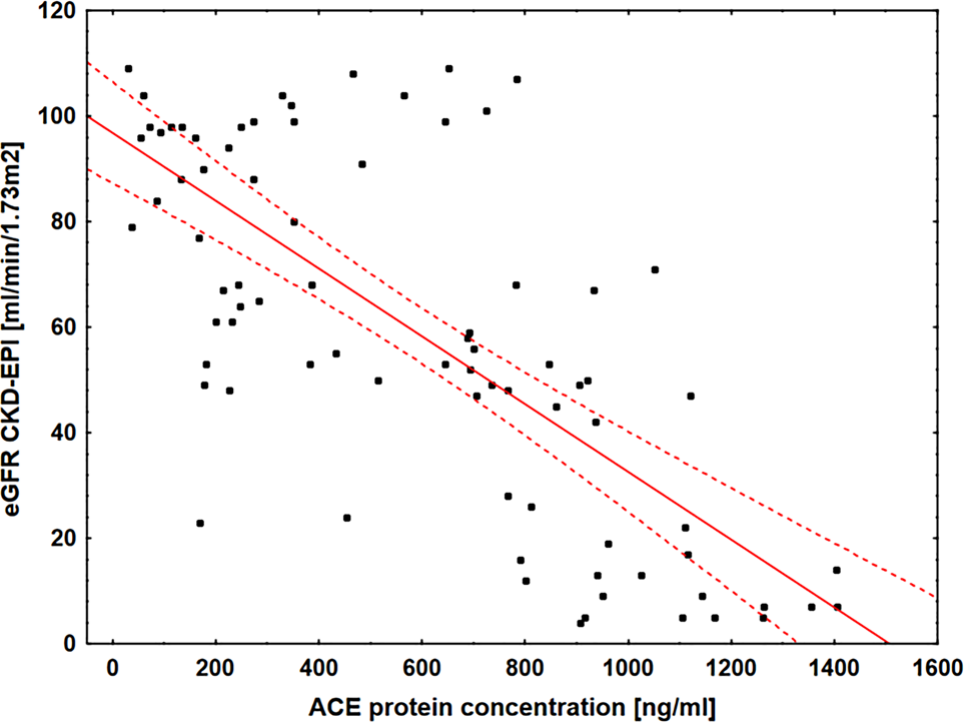
Scatterplot illustrating the linear negative correlation between serum ACE protein concentration and eGFR calculated using the CKD-EPI equation. The regression line with 95% confidence intervals (dashed lines) indicates a significant inverse association between ACE levels and eGFR across the study population. Abbreviations: ACE – angiotensin-converting enzyme; eGFR – estimated glomerular filtration rate; CKD-EPI – Chronic Kidney Disease Epidemiology Collaboration equation

A significant negative linear correlation was observed between serum ACE levels and eGFR (CKD-EPI formula), as shown in Fig. 4.

### DKD phenotype comparison

#### Clinical features

Only patients with the albuminuric phenotype (F1) required dialysis and had the highest prevalence of diabetic retinopathy. Macrovascular complications were significantly less frequent in the non-albuminuric phenotype F2. All patients in phenotype F3 had hypertension. Coronary heart disease was most prevalent in phenotypes F1 and F3, while it occurred much less frequently in F2. Stroke was observed exclusively in phenotypes F1 and F2. Statistically significant differences between phenotypes were found for both coronary heart disease (*p* = 0.001) and stroke (*p* = 0.03). Clinical data across phenotypes are summarized in Table 4.

**Table 4:** Clinical data of the study group divided into three DKD phenotypes

Parameter	F1 (*n* = 24)	F2 (*n* = 39)	F3 (*n* = 16)	*P*
Dialysis				
Yes No	8 (33.3%) 16 (66.7%)	0 (0%) 39 (100%)	0 (0%) 16 (100%)	< 0.00001
Retinopathy				
Yes No	10 (41.7%) 14 (58.3%)	1 (2.6%) 37 (97.4%)	0 (0%) 16 (100%)	0.00002
Coronary heart disease				
Yes No	12 (50%) 12 (50%)	3 (7.7%) 36 (92.3%)	7 (46.7%) 8 (53.3%)	0.001
Stroke				
Yes No	5 (20.8%) 19 (79.2%)	2 (5.1%) 37 (94.8%)	0 (0%) 16 (100%)	0.03
Hypertension				
Yes No	24 (100%) 0 (0%)	31 (79.5%) 8 (20.5%)	16 (100%) 0 (0%)	0.009
Amputation				
Yes No	5 (20.8%) 19 (79.2%)	0 (0%) 39 (100%)	1 (6.8%) 14 (93.2%)	0.005
Atherosclerosis				
Yes No	20 (83.3%) 4 (16.7%)	7 (17.9%) 32 (82.1%)	11 (73.3%) 4 (26.7%)	< 0.00001
Heart failure				
Yes No	10 (41.7%) 14 (58.3%)	3 (7.7%) 36 (92.3%)	8 (53.3%) 7 (46.7%)	0.0003
Death				
Yes No	6 (25%) 18 (75%)	0 (0%) 39 (100%)	1 (6.7%) 14 (93.3%)	0.002

Abbreviations: DKD – diabetic kidney disease

#### Laboratory and demographic characteristics

There were no significant differences between DKD phenotypes in BMI, HbA1c, VEGF-A, triglycerides (TG), low-density lipoprotein cholesterol (LDL-C), or UNCR. F1 patients had the highest ACE and lowest high-density lipoprotein cholesterol (HDL-C); fewest were women. F2 patients had the best kidney function (eGFR > 60 ml/min/1.73 m²), lowest AIP (0.41 ± 0.21), lowest ACE and hsa-miR-126-3p, and the highest proportion of women. F3 patients were oldest, had the highest uric acid, and borderline-high hsa-miR-423-5p; women predominated, but less so than in F2. Demographic and laboratory data are presented in Table 5.

**Table 5:** The demographic and laboratory data of the subjects in the present study divided into three DKD phenotypes. Data are presented as mean ± standard deviation or median (minimum–maximum). The letter designations A, B and C for the post-hoc multiple comparison of average ranks (medians) indicate differences between groups; * protein concentration; # Kruskal–Wallis test; ^ Chi² test

Parameter	F1	F2	F3	*p* ^#^
Age [year]	70.8 ± 9.6; 71.5 ^A^	60.7 ± 10.4; 61 ^B^	75.8 ± 8,4; 75 ^A^	< 0.0001
Percentage and number of patients [%(n)]	30.4 (24)	49.4 (39)	20.2 (16)	-
Gender – woman [n (%)]	9 (37.5%)^A^	27 (69.2%) ^B^	12 (75%) ^B^	0.02 ^
BMI [kg/m^2^]	29.2 ± 5.2; 28.5	32.5 ± 5.9; 33	32.1 ± 6.4; 30.2	0.1
ACE *[ng/ml]	966.3 ± 303; 955.6 ^A^	325.7 ± 261.5; 246.3 ^B^	628.5 ± 274.6; 692 ^C^	< 0.0001
VEGF-A * [pg/ml]	460.9 ± 384.4; 381.6	461 ± 359.7; 346.9	451.0 ± 157.2; 443.6	0.8
hsa-mir-126-3p [amol/µl]	0.6 ± 0.1; 0.6 ^A^	0.5 ± 0.1; 0.4 (0.4–0.9) ^AB^	0.5 ± 0.1; 0.4 ^B^	0.05
hsa-mir-423-5p [amol/µl]	0.2 ± 0.1; 0.2 ^A^	0.3 ± 0.1; 0.4 ^B^	0.3 ± 0.1; 0.4 ^AB^	0.05
Serum creatinine [mg/dl]	4.7 ± 2.7; 4 ^A^	0.8 ± 0.1; 0.7 ^B^	1.1 ± 0.2; 1.1 ^C^	< 0.0001
eGFR _CKD−EPI_ [ml/min/1.73m^2^]	17.4 ± 14.9; 13 ^A^	89.2 ± 16.3; 96 ^B^	51.1 ± 4.0; 50.5 ^A^	< 0.0001
HbA1c [%]	6.4 ± 0.7; 6.5	7.2 ± 2; 6.5	6.7 ± 1; 6.3	0.6
Uric acid [mg/dl]	5.9 ± 1.3; 5.9 ^A^	5.4 ± 1.2; 5.2 ^A^	6.6 ± 1.2; 6.6 ^B^	0.008
Total cholesterol [mg/dl]	164.3 ± 57.6; 147	185.1 ± 45.9; 174	164.3 ± 40.3; 160	0.06
LDL [mg/dl]	93.8 ± 41.6; 91.5	102.6 ± 41.6; 90	88.2 ± 32.6; 85	0.5
HDL [mg/dl]	40.6 ± 9.7; 40 ^A^	51.8 ± 13.1; 50 ^B^	46.5 ± 8.9; 49 ^AB^	0.001
TG [mg/dl]	164 ± 104.3; 131	142 ± 61.1; 117	148.0 ± 71.7; 119.5	0.9
AIP	0.54 ± 0.25; 0.54	0.41 ± 0.21; 0.38	0.46 ± 0.24; 0.43	0.1
UACR [mg/g]	281.2 ± 63.7; 300 ^A^	7 ± 6.3; 5 ^B^	7.9 ± 7.5; 6.3 ^B^	< 0.0001
UNCR [ug/g]	50.1 ± 30.3; 56.9	17.5 ± 15.2; 11.2	31.8 ± 29.2; 23.3	0.06

Abbreviations: ACE – angiotensin-converting enzyme; AIP – atherogenic index of plasma; BMI – body mass index; DKD – diabetic kidney disease; eGFR – estimated glomerular filtration rate; HbA1c – glycated hemoglobin; HDL-C – high-density lipoprotein cholesterol; hsa-miR – human microRNA; LDL-C – low-density lipoprotein cholesterol; TG – triglycerides; UACR – urinary albumin-to-creatinine ratio; UNCR – urinary nephrin-to-creatinine ratio; VEGF-A – vascular endothelial growth factor A

#### Subgroup analysis: phenotype F4 (moderately increased albuminuria, UACR 30–300 mg/g)

This exploratory subgroup was not included in the primary F1–F3 comparisons. Compared across phenotypes, F4 showed preserved kidney function (*p* < 0.0001), the lowest BMI (*p* = 0.02), the highest HDL-C (*p* = 0.001), intermediate ACE (lower than F1/F3, higher than F2; *p* < 0.0001) and higher uric acid (*p* = 0.01). Differences in AIP, VEGF-A, UNCR and the circulating miRNAs (hsa-miR-126-3p, hsa-miR-423-5p) were not statistically significant. Clinically, there were no cases of dialysis, retinopathy, amputations or death, whereas macrovascular burden remained non-trivial: coronary heart disease and stroke differed significantly across phenotypes (*p* = 0.003 and *p* = 0.02, respectively).

**Fig. 5:**
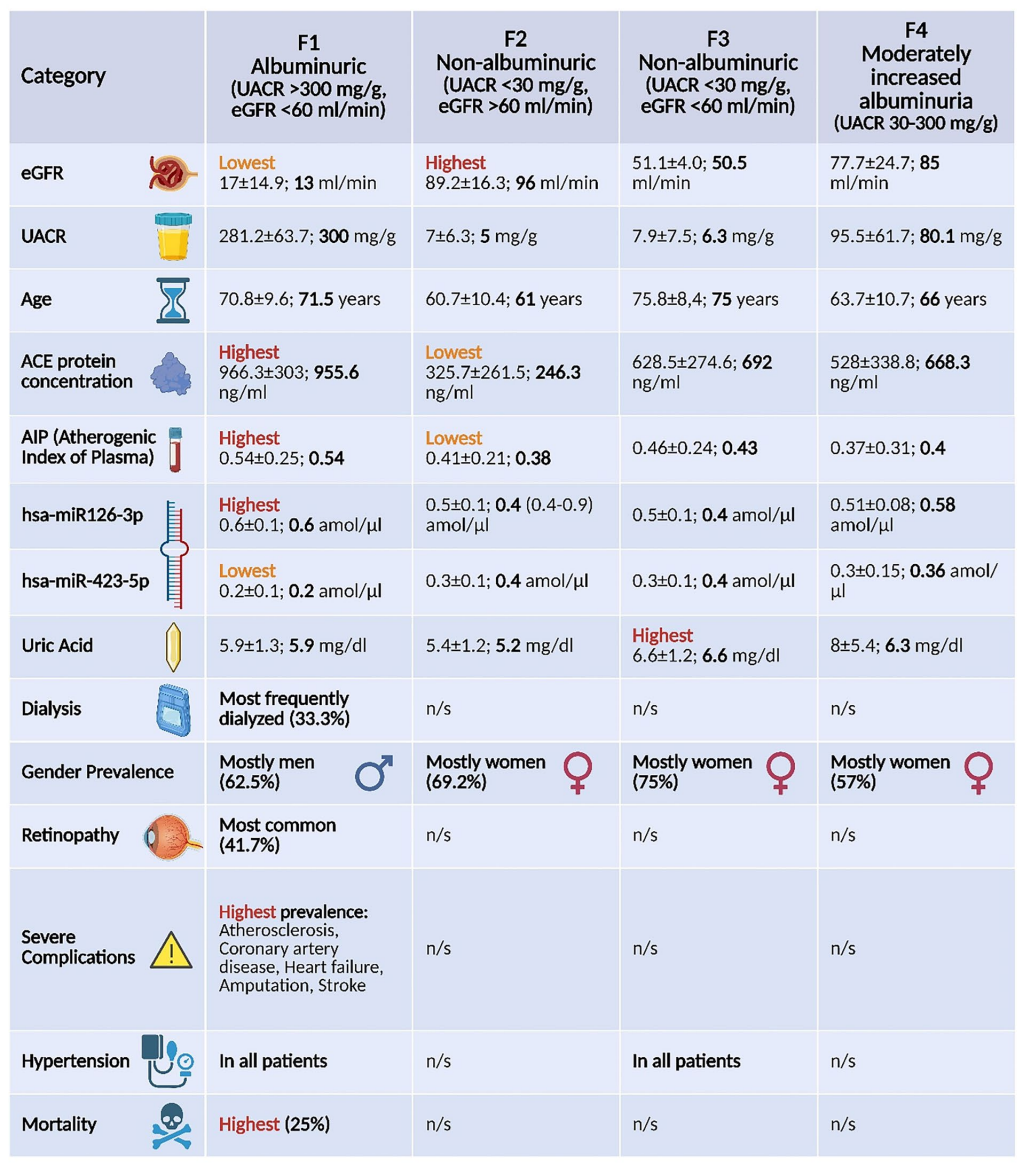
Baseline clinical, demographic, and molecular features across DKD phenotypes F1–F4. Continuous variables are shown as mean ± SD; categorical variables as percentages. Boldface marks the within-row extreme (highest or lowest); where font color is additionally used, red denotes the highest value and orange the lowest. “n.s.” denotes no statistically significant differences. F4 (KDIGO A2, 30–300 mg/g) is presented descriptively only and was excluded from primary comparisons and from the final discriminant model. Abbreviations: eGFR — estimated glomerular filtration rate (CKD-EPI); UACR — urinary albumin-to-creatinine ratio; AIP — atherogenic index of plasma; ACE — angiotensin-converting enzyme; miR — microRNA; n.s. — not significant. (Created with BioRender.com.)

A summary of clinical, demographic and molecular features across F1–F4 is presented in Fig. 5.

### Discriminant analysis

Discriminant analysis used age, ACE, uric acid, and AIP.

Group F4 was not included in the final discriminant analysis because its inclusion substantially reduced classification accuracy and interpretability. The F4 subgroup—patients with moderately increased albuminuria and preserved eGFR—formed a heterogeneous, poorly separable cluster (correct classification 30.0%; four-group overall accuracy 75.8%), thereby weakening both statistical robustness and clinical clarity. We therefore restricted the analysis to the prespecified core phenotypes F1–F3, for which the method yielded clearer clinical contrasts and stronger performance.

Two functions were derived:

- D1=-3.97-0.008*age + 0.004*ACE + 0.19*uric acid + 1.86*AIP.
- D2=-7.89 + 0.11*age-0.002*ACE + 0.32*uric acid-0.67*AIP.

**Fig. 6:**
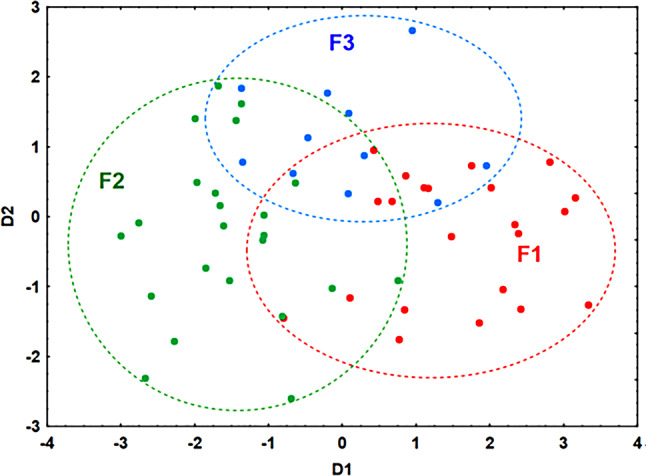
The classification results were visualized using a scatterplot showing the distribution of observations within the space defined by the two discriminant functions (D1 and D2). This visualization effectively illustrates the separation between the F1, F2, and F3 groups and supports the accuracy of the discriminant model. The findings emphasize the importance of uric acid and API as key variables influencing group differentiation. Abbreviations: D1 – first discriminant function; D2 – second discriminant function; AIP – Atherogenic Index of Plasma; DKD – diabetic kidney disease

AIP and uric acid had the greatest influence. Classification accuracy was 87.5%, with Wilks’ Lambda *p* < 0.05 confirming statistical significance. Discriminant classification results are presented in Fig. 6.

### miRNA expression in DKD phenotypes

**Fig. 7:**
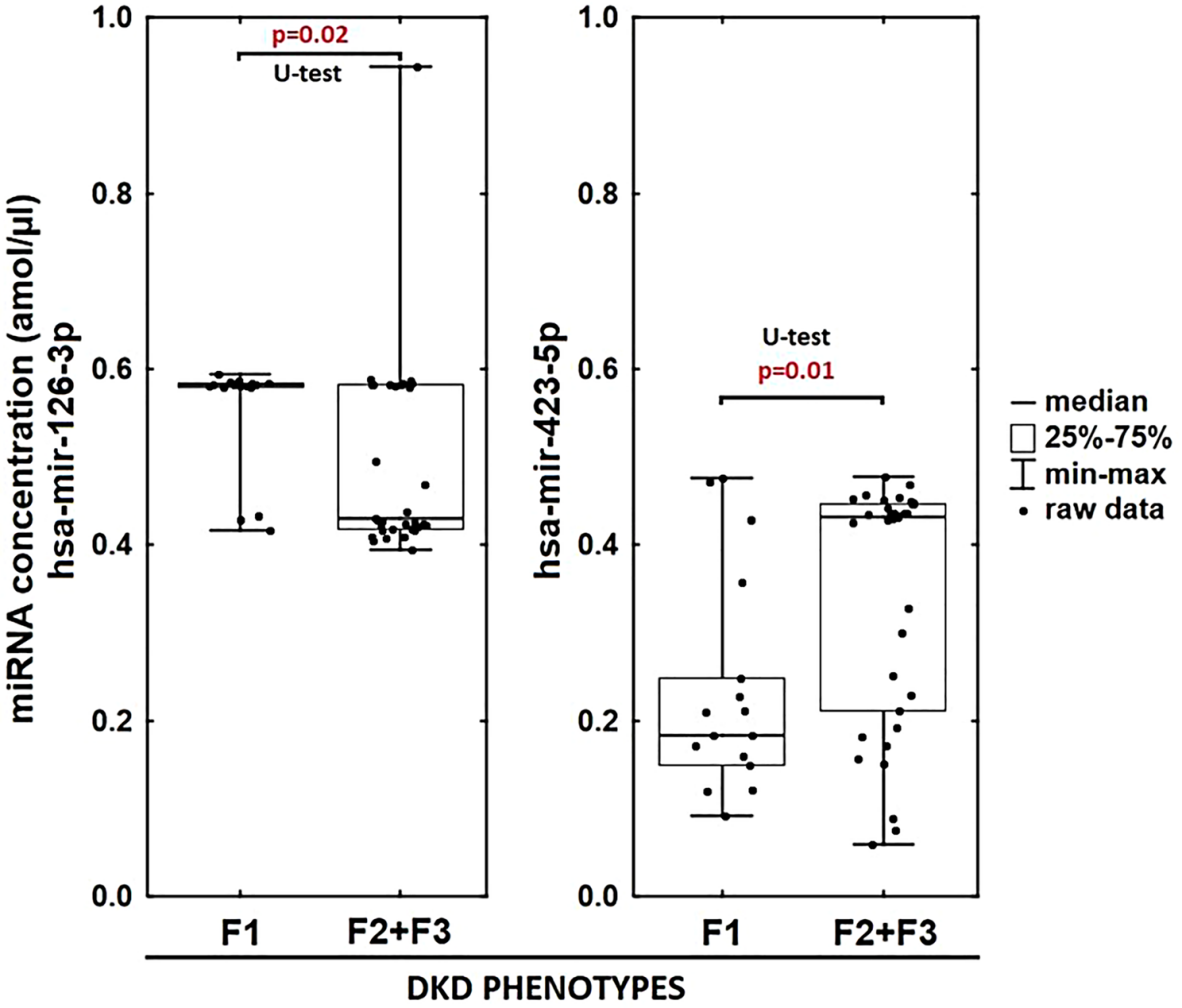
Differential expression of hsa-miR-126-3p and hsa-miR-423-5p across DKD phenotypes. The box plot illustrates the concentration of two microRNAs, hsa-miR-126-3p and hsa-miR-423-5p, expressed in attomoles per microliter (amol/µl), across different DKD phenotypes. The left panel represents hsa-miR-126-3p concentration in two groups: F1 and F2 + F3. The median, interquartile range (25%–75%), and min–max values are shown, along with individual raw data points. A U-test was performed, indicating a statistically significant difference (*p* = 0.02) between the two groups. The right panel depicts hsa-miR-423-5p concentration following the same grouping. A U-test also revealed a significant difference (*p* = 0.01) between the groups. The results suggest differential expression of these miRNAs in distinct DKD phenotypes, which may have potential implications for disease progression or biomarker discovery. Abbreviations: DKD – diabetic kidney disease; F1 – albuminuric phenotype; F2 + F3 – non-albuminuric phenotypes; hsa-miR – human microRNA; U-test – Mann–Whitney U test

hsa-miR-126-3p expression was significantly higher in F1 than F2 + F3 (*p* = 0.02; Mann-Whitney U test), while hsa-miR-423-5p was significantly higher in F2 + F3 (*p* = 0.01), with greater interindividual variability. Detailed miRNA expression distributions are shown in Fig. 7.

## Discussion

This study provides new evidence on the phenotypic heterogeneity of DKD and identifies ACE, AIP, and circulating miRNAs (miR-126-3p and miR-423-5p) as potential biomarkers of kidney function decline in patients with T2DM. The use of phenotype-based classification (F1–F3), incorporating potential biomarkers in correlation with clinical data and PLS-SEM modeling, enabled a better understanding of the underlying pathogenic mechanisms across different DKD subgroups [1].

For completeness, we also depict the intermediate subgroup with UACR 30–300 mg/g (F4) in Figs. 2 and 5. In the discriminant space, this subgroup was heterogeneous and poorly separable, reducing the overall accuracy of a four-group model to 75.8% (30% correct classification for F4). We therefore report the discriminant analysis within the prespecified F1–F3 framework, which yielded clearer clinical contrasts and higher performance.

### Determinants of kidney function in DKD

ACE, AIP and age were associated with lower eGFR, consistent with prior work implicating inflammation, dyslipidemia and RAAS activation in DKD progression [9–16]; male sex also showed adverse associations in univariable analyses but did not remain an independent predictor after adjustment. In the multivariable model, ACE, AIP and age remained independent negative predictors, whereas miR-423-5p emerged as a positive predictor of eGFR. The relationship between miR-126-3p, ACE and eGFR aligns with reports linking this miRNA to endothelial stress and glomerular dysfunction [12]. Consistent with the correlation analysis, miR-423-5p showed a modest positive association with eGFR and an inverse association with UACR, while miR-126-3p tracked with UACR only; these small but directionally consistent associations indicate complementary information about renal function and albuminuria.

### RAAS activation and phenotype-dependent ACE profile

A key finding of this study is the strong inverse linear correlation between ACE concentration and eGFR, regardless of DKD phenotype. ACE levels were highest in the F1 (albuminuric) group, intermediate in F3, and lowest in F2, suggesting a variable degree of RAAS activation depending on phenotype. These results are consistent with previous studies conducted in small cohorts [18] and extend earlier observations by Ustündağ et al. from 2000 [19], indicating the potential use of ACE as a phenotype-specific biomarker of DKD progression.

From a pathophysiological perspective, elevated ACE reflects enhanced conversion of angiotensin I to angiotensin II, which exerts pro-inflammatory, pro-oxidative, and profibrotic effects [20]. Thus, high ACE may serve not only as a diagnostic marker but also as a potential target for individualized therapy, particularly in the F1 phenotype.

An additional aspect that should be considered is the potential influence of RAAS inhibitor therapy on circulating ACE concentrations. In our study, 59.5% of patients (47/79) received ACE inhibitors or ARBs, with no significant differences between phenotypes (50.0% in F1, 60.5% in F2, and 70.6% in F3; *p* = 0.4). Serum ACE levels are not reduced by ACE inhibitors; in most studies they remain unchanged, and in some cases they even increase, a phenomenon referred to as ACE escape [18, 20]. Similarly, ARBs do not directly inhibit ACE and, through feedback mechanisms, may contribute to its upregulation. Furthermore, genetic polymorphisms may modulate ACE concentrations and treatment response [15]. Therefore, the phenotype-dependent ACE gradient observed in our study (highest in F1, lowest in F2, intermediate in F3) is unlikely to result solely from treatment differences but rather reflects intrinsic RAAS activation related to the pathophysiology of each phenotype [18].

Another important point is the overall prevalence of RAAS inhibitor therapy in our cohort. Although 59.5% of patients were treated with ACE inhibitors or ARBs, this figure may appear modest compared with guideline recommendations. Nevertheless, it reflects real-world clinical practice in advanced CKD, where contraindications such as hyperkalemia, symptomatic hypotension, progressive decline in kidney function, or documented drug intolerance often limit their use. Similar findings have been consistently reported in population-based studies, which showed a substantial decline in RAAS inhibitor use among patients with low eGFR compared to earlier CKD stages [21, 22]. This discrepancy illustrates the well-recognized evidence–practice gap in nephrology, with implementation science studies suggesting that it often takes more than a decade before guideline-directed therapies are fully adopted into routine care [23].

### miRNA expression and DKD phenotype

This study is the first to demonstrate that miR-126-3p is significantly overexpressed in the albuminuric DKD phenotype (F1), whereas miR-423-5p predominates in non-albuminuric phenotypes (F2 and F3). These differences suggest distinct underlying mechanisms: endothelial injury and compensatory angiogenesis in F1, versus tubulointerstitial fibrosis and oxidative stress in F2/F3. This supports the concept of phenotypic diversity in DKD and highlights the potential utility of miRNA profiling in differential diagnosis [24].

### Sex-related differences in DKD pathophysiology

Male patients were more frequently represented in the F1 phenotype and exhibited higher levels of ACE and AIP, suggesting enhanced RAAS activation and metabolic stress. Female patients - particularly those in the F2 phenotype - had lower ACE and miR-126-3p levels and better kidney function, consistent with the protective role of estrogens [25]. These findings underscore the importance of sex as a biological factor influencing the course of DKD.

### Clinical and molecular factors associated with mortality in the DKD population

Among the variables analyzed, higher ACE concentration, older age, lower eGFR, albuminuric phenotype F1, and elevated AIP appeared to be significantly associated with mortality in the studied population. These findings highlight the prognostic value of parameters reflecting RAAS activation, metabolic dysfunction, and DKD progression. While these observations are not novel [10, 26, 27], they support the representativeness of the study cohort. Identification of such factors may aid in the early recognition of patients who require intensified nephroprotective and cardiometabolic interventions.

### Cardiovascular burden across DKD phenotypes

Coronary heart disease was significantly more prevalent in DKD phenotypes F1 and F3 compared to F2. Stroke occurred only in F1 and F2. These findings suggest that macroangiopathic risk is not restricted to albuminuric phenotypes and support the clinical relevance of cardiovascular risk stratification across DKD phenotypes.

### Strengths, limitations, and research perspectives

Strengths of this study include predefined clinical phenotyping (F1–F3) using clear eGFR/UACR thresholds, inclusion of a descriptive F4 subgroup for completeness, integration of blood biochemistry with circulating miRNA profiling, inclusion of dialysis patients, and the use of advanced analytical methods (PLS-SEM and discriminant modeling). Limitations are the modest sample size, the absence of protocol renal biopsies (precluding gold-standard, histology-based severity stratification and direct linkage of miRNA profiles to specific lesions), and the lack of formal assessment of treatment effects (e.g., SGLT2 inhibitors) on biomarker levels. The F4 subgroup (UACR 30–300 mg/g) was analyzed exploratorily and excluded from primary comparisons and the discriminant model because of low class separability, which may limit generalizability to early DKD. Future studies should validate these observations in larger, biopsy-characterized cohorts; incorporate longitudinal sampling to test treatment effects; evaluate broader biomarker panels (e.g., tubular/inflammatory markers); and explore non-linear or ensemble classifiers to better capture heterogeneous early-stage phenotypes and to advance biomarker-guided, personalized therapy (e.g., ACE, miR-126-3p, miR-423-5p).

## Conclusions and clinical implications

The findings of this study confirm the significant heterogeneity of DKD in the course of T2DM and highlight the utility of molecular biomarkers (hsa-miR-126-3p, hsa-miR-423-5p), metabolic indices (AIP), and ACE concentration in differentiating clinical DKD phenotypes. The identified molecular and clinical associations have direct relevance to nephrology practice:

- ACE and miR-126-3p may serve as early markers of disease progression, particularly in albuminuric phenotypes (F1), simultaneously indicating RAAS activation as a potential therapeutic target.
- miR-423-5p may serve as an indicator of non-albuminuric phenotypes (F2–F3) associated with tubulointerstitial injury, suggesting a distinct pathogenesis that may require alternative treatment strategies.
- Phenotype-based classification of DKD (F1–F3) demonstrated significant prognostic and discriminatory value, supporting the need for personalized nephroprotective therapy tailored to the patient’s molecular profile.
- The use of PLS-SEM modeling enabled the identification of complex interactions between biomarkers and clinical patient characteristics, offering a potential predictive tool in clinical practice.
- The observed differences in the prevalence of coronary heart disease and stroke between phenotypes indicate that cardiovascular risk may be underestimated in patients without albuminuria, supporting the need for systematic risk assessment regardless of classical DKD markers.
- The study highlights sex-related differences in DKD phenotype distribution and biomarker expression, supporting the role of biological sex as a factor influencing disease progression.

Thus, early identification of the DKD phenotype using the proposed biomarkers may improve risk stratification and allow for the implementation of therapies targeting the predominant pathogenic mechanisms rather than merely addressing clinical symptoms.

## Data Availability

All data related to this study are available from the corresponding author upon reasonable request.

## Abbreviations

ACE: Angiotensin-converting enzyme
AIP: Atherogenic index of plasma
ARBs: Angiotensin receptor blockers
ASA: Acetylsalicylic acid
BMI: Body mass index
CKD: Chronic kidney disease
CKD-EPI: Chronic Kidney Disease Epidemiology Collaboration
DKD: Diabetic kidney disease
DM: Diabetes mellitus
DN: Diabetic nephropathy
eGFR: Estimated glomerular filtration rate
ELISA: Enzyme-linked immunosorbent assay
HbA1c: Glycated hemoglobin
HDL-C: High-density lipoprotein cholesterol
hsa-miR: Human microRNA
KDIGO: Kidney Disease: Improving Global Outcomes
LDL-C: Low-density lipoprotein cholesterol
miRNA: microRNA
PLS-SEM: Partial least squares structural equation modeling
RAAS: Renin–angiotensin–aldosterone system
SRMR: Standardized root mean square residual
T1DM: Type 1 diabetes mellitus
T2DM: Type 2 diabetes mellitus
TG: Triglycerides
UACR: Urinary albumin-to-creatinine ratio
uEVs: urinary extracellular vesicles
UNCR: Urinary nephrin-to-creatinine ratio
VEGF-A: Vascular endothelial growth factor A
